# Unsupervised Learning of Monocular Depth and Ego-Motion with Optical Flow Features and Multiple Constraints

**DOI:** 10.3390/s22041383

**Published:** 2022-02-11

**Authors:** Baigan Zhao, Yingping Huang, Wenyan Ci, Xing Hu

**Affiliations:** 1 School of Optical-Electrical and Computer Engineering, University of Shanghai for Science & Technology, Shanghai 200093, China; 171560051@st.usst.edu.cn (B.Z.); huxing@usst.edu.cn (X.H.); 2 School of Engineering, Huzhou University, Huzhou 313000, China; 02849@zjhu.edu.cn

**Keywords:** unsupervised learning, depth recovery, ego-motion estimation, optical flow

## Abstract

This paper proposes a novel unsupervised learning framework for depth recovery and camera ego-motion estimation from monocular video. The framework exploits the optical flow (OF) property to jointly train the depth and the ego-motion models. Unlike the existing unsupervised methods, our method extracts the features from the optical flow rather than from the raw RGB images, thereby enhancing unsupervised learning. In addition, we exploit the forward-backward consistency check of the optical flow to generate a mask of the invalid region in the image, and accordingly, eliminate the outlier regions such as occlusion regions and moving objects for the learning. Furthermore, in addition to using view synthesis as a supervised signal, we impose additional loss functions, including optical flow consistency loss and depth consistency loss, as additional supervision signals on the valid image region to further enhance the training of the models. Substantial experiments on multiple benchmark datasets demonstrate that our method outperforms other unsupervised methods.

## 1. Introduction

Depth recovery and camera ego-motion estimation from monocular video are fundamental topics in computer vision with numerous applications in industry, including robotics, driverless vehicles, and navigation systems. Traditional solutions to these tasks rely on binocular stereo techniques or structure-from-motion methods, which reconstruct 2D images into the 3D world by analyzing the geometric difference between left–right or/and consecutive images [1]. Camera ego-motion, also known as visual odometry (VO), is the process of calculating an agent’s pose solely based on images captured by a single or multiple cameras mounted to it. The basic VO framework follows a standard pipeline, which typically includes feature detection, feature tracking, outlier rejection, motion estimation and optimization [2,3,4]. Although these methods are accurate and robust under favorable conditions, they are sensitive to camera parameters and are more unstable in extreme environments, such as textureless areas and lighting changes.

Recently, convolutional neural networks (CNNs) have become increasingly popular in computer vision tasks, providing researchers with a new solution to depth recovery and ego-motion estimation. Learning-based methods can be classified into two groups including supervised and unsupervised methods in terms of whether they rely on ground truth for training. Supervised methods learn the functions to map the depth and ego-motion to the image by minimizing the differences between the estimated values and the related ground truth [5,6,7,8,9,10,11,12,13,14,15]. However, supervised methods need a massive quantity of ground truth data to train the model, which is both costly and difficult to get in reality. Moreover, the dependency on ground truth data leads to unsatisfactory performance in new environments or some unlearned scenarios.

Instead of using expensive ground truth data, unsupervised methods train CNN models directly using unlabeled data, thereby saving human effort on data-labeling, allowing the use of a larger amount of data for training, and achieving better generalization. A common principle of the existing unsupervised methods [16,17,18,19,20,21,22,23] is to train the CNN models by using a synthesized view as a supervisory signal. We call it the view synthesis technique, where one view (source) is synthesized into another (target) based on the estimated camera ego-motion and the predicted depth of the target view. The unsupervised framework is subsequently trained by decreasing the photometric difference between the synthesized and original target views. Three issues exist in regard to the existing unsupervised methods: (1) The synthesized view is subject to error because these works do not apply optical flow (OF) to estimate camera ego-motion but directly use RGB images, which contain complex and redundant information. In fact, the OF field implies geometric motion between consecutive images and is a key factor for accurate view synthesis and ego-motion estimation. Our previous work [14] and the authors of [13,15] have demonstrated that OF is highly effective for learning VO. (2) These methods conduct the learning on the whole area of the synthesized image containing substantial outliers such as occluded regions and moving objects, which can inhibit the training of the network and cause a significant error in the results. (3) These methods design loss function by considering the photometric consistency between the synthesized and original target views, which provides a weak constraint for unsupervised learning and thus may generate inaccurate training results.

To overcome these disadvantages, this paper proposes a new unsupervised framework for improving unsupervised learning and the performance of depth recovery and camera ego-motion estimation. The framework learns the depth and VO models from the accurate OF field rather than from the raw RGB images. Moreover, we exploit the forward-backward consistency check of the optical flow to generate a mask of the invalid region in the image, and accordingly, eliminate outlier regions such as occlusion regions and moving objects for the learning. Furthermore, we adopt several constraints for defining the multiple loss functions to further enhance the unsupervised learning. In brief, the following are our main contributions:

1. We propose a novel unsupervised learning framework for estimating the depth and camera ego-motion. By virtue of the optical flow property, the framework extracts the features from the optical flow rather than from the raw RGB images, thereby enhancing unsupervised learning;
2. We eliminate the outlier regions such as occlusion regions and moving objects for the learning by generating a mask of the invalid region in the scene according to the forward-backward consistency of the optical flow, thereby preventing the training from being inhibited and improving the performance;
3. We propose optical flow consistency loss and depth consistency loss as additional supervision signals to further enhance the training of the models;
4. We conduct extensive experiments on multiple benchmark datasets, and the results demonstrate that our method outperforms the existing unsupervised algorithms.

## 2. Related Work

We primarily discuss related works that use machine learning methods for separate or joint learning of depth and ego-motion. As mentioned above, learning-based methods can be divided into two groups, including supervised and unsupervised methods in terms of whether they rely on ground truth for training. 

### 2.1. Supervised Learning of Monocular Depth and Ego-Motion

Existing supervised learning-based works normally treat depth recovery and ego-motion as two separate tasks and conduct learning for each goal by minimizing the differences between the estimated values and the related ground truth.

The authors of [5] were the first to predict depth from a single image via supervised learning. They accomplished this work by combining two deep network stacks: one that generates a rough global prediction based on the full image and another that rectifies this prediction locally. Different from [5], which employs an extra network to improve the results, Liu et al. [6] presented an approach based on the hierarchical conditional random fields (CRFs) to enhance the depth map. Meanwhile, they proposed a super-pixel pooling method to accelerate convolutional networks. Recently, several works [7,8,9] have used adversarial learning to estimate depth and have proven to be beneficial.

DeepVO [10] is a typical supervised learning method to estimate camera motion. CNN was utilized to learn effective feature representation, while an RNN was employed to describe sequential dynamics and connections. DeepVO completed an end-to-end pose estimation and obtained competitive accuracy and generalization ability. Based on this typical model, several studies expanded on this strategy to increase model performance. In [11], the authors considered the curriculum learning (CL) technique (training a model by gradually increasing the complexity of the training data) to increase the generalization capacity of supervised VO. In [12], knowledge distillation (transferring the knowledge of a huge teacher model to a small student model) was used in the supervised VO framework to drastically decrease the amount of network parameters, making it more suitable for real-time operation on portable devices. Since the OF field implies geometric motion, learning optical flow for VO is a common technique for learning-based VO methods such as in [13,14,15]. In [13], the authors proposed to use an auto-encoder network to find a nonlinear representation of the OF field for ego-motion estimation. Our previous work [14] further proved that learning the latent space of the OF field is effective for ego-motion estimation. We conducted sequential learning by using the RCNN network to regress the OF latent space into the 6-DOF camera ego-motion. In [15], Zhao et al. not only used OF field to estimate the camera ego-motion, but also investigated the capacity of deep neural networks for state estimation to filter the 6-DOF trajectory given a sequence of measurements.

Since the supervised approaches are guided by the ground truth, they can effectively train the functions to map the depth and ego-motion to the image and have produced outstanding results. However, these supervised algorithms are constrained by labeled datasets, which are difficult and expensive to obtain and may be short of generalization.

### 2.2. Unsupervised Learning of Monocular Depth and Ego-Motion

Existing unsupervised learning-based works normally conduct joint learning on depth and ego-motion simultaneously by using true constraints as supervisory signals for training. Since the depth recovery and camera pose estimation are closely connected in terms of their internal geometric relationship, the main supervisory signal can be obtained by jointly training these tasks in the lack of ground truth and stereo frames. The existing unsupervised methods [16,17,18,19,20,21,22,23] adopt the synthesized view as a supervisory signal to train the models.

A typical unsupervised method was proposed by Zhou et al. [16] that utilizes view synthesis as a supervised signal to jointly learn depth and camera pose from image sequences. Specifically, this framework is made up of two networks: a depth network for estimating depth and a pose network for calculating camera ego-motion. Based on the predicted depth and the estimated camera ego-motion, one view (source) can be synthesized into another (target). The CNN models are then trained by minimizing the photometric differences between the synthesized and original target views. Motivated by this basic model, several studies [17,18,19,20,21,22,23] have been done to improve on it and achieved good results. Aiming at the scale problem, Zhan et al. [17] recovered the absolute scale using stereo image pairs. Meanwhile, they introduced a feature reconstruction loss to increase depth and pose estimation accuracy. In [18], Mahjourian et al. introduced an ICP loss to ensure the consistency of the calculated 3D point clouds between the consecutive frames. They trained the network using a combination of 3D and 2D losses and achieved good results. In [19], Yang et al. introduced edge estimation, which improves the performance of the model by jointly estimating the edge and the 3D scene. In [20], Jiang et al. introduced an outlier masking strategy that treats occluded or dynamic pixels as statistical outliers, hence avoiding the negative impacts of occlusion and dynamics on learning in realistic environments.

Recently, several studies have taken advantage of the inherent geometric connection between depth, ego-motion, and optical flow to jointly train the models of these subtasks. In [21], the authors introduced GeoNet, a collaborative learning framework for estimating depth, ego-motion, and optical flow. They used an additional network to learn the residual optical flow for the scene’s dynamic objects. As a result of segregating rigid and non-rigid scenes, the accuracy of all three estimations was enhanced. Instead of estimating residual optical flow, Zhang et al. [22] added an extra network to predict the optical flow. They also introduced multi-view consistency losses to constrain the framework for better performance. Ranjan et al. [23] enhanced the multi-task framework by incorporating a motion segmentation task based on the results of other tasks (depth recovery, pose estimation and optical flow estimation). The increase in the number of tasks makes the training more complicated, so they introduced competitive collaboration, a framework for coordinating the training of various specialized neural networks to address complicated tasks. In these multi-task-based methods, OF estimation is added as a subtask network and trained alongside the depth and pose networks. The errors caused by the depth and pose calculation will inevitably be transmitted to the optical flow, and optical flow with poor accuracy will in turn affect the learning of the depth and pose.

## 3. Methods

**Figure 1:**
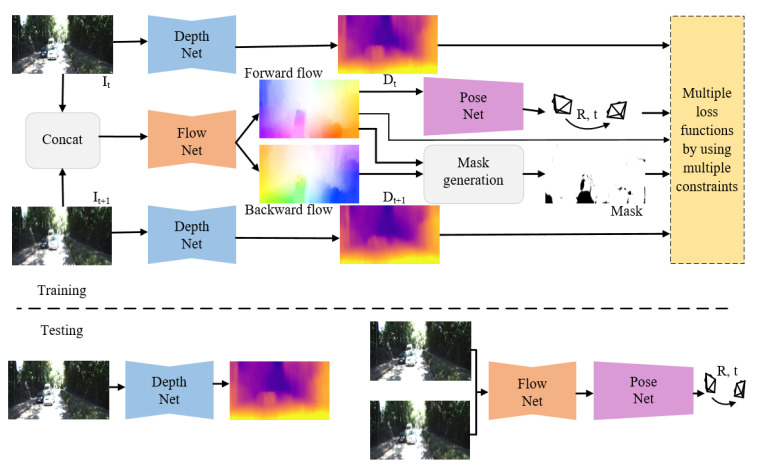
Overview of our method. During training, we use the unlabeled image sequences as the input of the network. FlowNet is an off-the-shelf optical flow estimation network to generate accurate OF fields. DepthNet and PoseNet are jointly trained for the prediction of the depth and pose. We exploit the forward-backward consistency check of the optical flow to mark the invalid region in the scene, so as to avoid the adverse effects of the outlier regions, such as occlusion regions and moving objects on training. Finally, we use multiple constraints as loss functions to train DepthNet and PoseNet. In the test stage, DepthNet and PoseNet are used to estimate the single-view depth and the 6-DOF camera pose separately.

The framework of the proposed method is shown in Figure 1. It is composed of three CNN networks: DepthNet, PoseNet and FlowNet (Section 3.1). An off-the-shelf optical flow estimation network was used as the FlowNet to generate accurate OF fields. Our goal was to jointly train the DepthNet and the PoseNet by using unlabeled monocular image sequences so that the two networks can estimate single-view depth and camera motion separately during testing. Given the consecutive images $\left(I_{t},\;I_{t+1}\right)$, we first estimated the depth of frame $I_{t}$ and $I_{t+1}$, and the forward-backward OF fields between frame $I_{t}$ and $I_{t+1}$. Then we used the forward OF field to estimate the camera pose between the consecutive images $\left(I_{t},\;I_{t+1}\right)$. With the predicted depth map and the estimated 6-DOF camera ego-motion, frame $I_{t+1}$ can be synthesized into frame $I_{t}$. The synthesized frame ${\bar{I}}_{t}$ and the original frame $I_{t}$ should be consistent in terms of photometry (Section 3.2). With the estimated forward-backward OF fields, we could generate a mask of the invalid regions in the image, and then eliminate the outliers such as occlusions and moving objects according to the forward-backward consistency of the optical flow (Section 3.3). In addition, the predicted depth map and the estimated camera pose can be used to calculate the OF field, which should be consistent with the generated OF field from FlowNet in the rigid area of the image (Section 3.4). Based on the generated forward OF field, the depth map $\left(D_{t+1}\right)$ can be synthesized into depth map $\left(D_{t}\right)$; the synthesized depth map $\left({\bar{D}}_{t}\right)$ should be consistent with the target depth map $\left(D_{t}\right)$ (Section 3.4). The objective function is defined by considering four constraints including photometric consistency, smoothness, optical flow consistency and depth consistency, and is formulated as

(1) $$L=\underset{l}{\sum }(L_{pho}^{l}+\lambda_{s}L_{smo}^{l}+\lambda_{f}L_{flo}^{l}+\lambda_{d}L_{dep}^{l})$$

where *l* indicates different image scales, $L_{pho}^{l}$, $L_{smo}^{l}$, $L_{flo}^{l}$ and $L_{dep}^{l}$ indicate photometric consistency loss, smoothness loss, optical flow consistency loss and depth consistency loss, respectively, and $\lambda_{s},\;\lambda_{f}\;\mathrm{and}\;\lambda_{d}$ represent their corresponding weights.

### 3.1. The Networks

The framework in Figure 1 contains three subnetworks: DepthNet, PoseNet and FlowNet. The FlowNet is an off-the-shelf optical flow estimation network to generate accurate OF fields. We used a fixed-weight MaskFlownet [24] as the FlowNet. We adopted the DispResNet [23], an encoder-decoder network with skip connections and multi-scale side predictions, as the DepthNet. For the PoseNet, we used the network proposed in [16]. The difference is that we used the optical flow of adjacent images as input to estimate the 6-DOF camera pose instead of directly using adjacent images.

### 3.2. Photometric Consistency Loss and Smoothness Loss

We used the view synthesis as the main supervision signal to jointly learn depth and camera motion from unlabeled video sequences. Given the consecutive images $\left(I_{t},\;I_{t+1}\right)$, the estimated depth map $\left(D_{t}\right)$ at time *t* and the estimated relative camera pose $\left(T_{t\to t+1}\right)$, we can establish the dense pixel correspondence between the consecutive images $\left(I_{t},\;I_{t+1}\right)$. When $p_{t}$ denotes the coordinate of a pixel in frame $I_{t}$, the corresponding point of $p_{t}$ in frame $I_{t+1}$ can be computed via:

(2) $$p_{t+1}\sim KT_{t\to t+1}D_{t}\left(p_{t}\right)K^{-1}p_{t}$$

where *K* indicates the camera’s intrinsic matrix. According to this geometric correspondence, we can synthesize a new image $\left({\bar{I}}_{t}\right)$ with the inverse warping from frame $\left(I_{t+1}\right)$. When the scene is static, there is no occlusion between the consecutive frames and the surface is Lambertian, the synthesized image $\left({\bar{I}}_{t}\right)$ should be consistent with the target image $\left(I_{t}\right)$. The photometric discrepancy between the synthesized image and the original image can be used as an unsupervised loss function for training CNNs. Specifically, the photometric consistency loss function can be formulated as:

(3) $$L_{pho}=\underset{p_{t}}{\sum }\rho \left(I_{t}\left(p_{t}\right)-{\bar{I}}_{t}\left(p_{t}\right)\right)$$

where $\rho \left(x\right)={\left(x^{2}+\epsilon^{2}\right)}^{\gamma }$ is the robust generalized Charbonnier penalty function with γ=0.45 and $\epsilon =10^{-3}$ [25]. In previous works [16,17,18], the loss function mostly used the combination of an L1 norm and a structural similarity (SSIM) to measure the photometric discrepancy, which is not suitable for realistic situations where illumination changes. This loss function [25] is used to compensate for additive and multiplicative illumination changes, thus providing us with a more reliable constancy assumption for realistic imagery.

Since the photometric consistency loss is not informative in the low-texture or homogeneous region of the scene, we adopted the edge-aware smoothness loss used in [21] to keep sharp details, which is formulated as:

(4) $$L_{smo}=\underset{p_{t}}{\sum }\left|\nabla D\left(p_{t}\right)\right|\cdot {\left(e^{-\left|\nabla I\left(p_{t}\right)\right|}\right)}^{T}$$

where |·| indicates element-wise absolute value, ∇ represents the vector differential operator, and *T* denotes the transpose of image gradient weighting.

### 3.3. Outlier Region Elimination

Under the premise of photometric consistency, the synthesized image should be photometrically compatible with the target image. However, this assumption does not hold for the outlier regions such as occlusion regions and moving objects. Therefore, we need to eliminate the outlier region in the scene and only impose the photometric consistency loss on the valid region.

**Figure 2:**
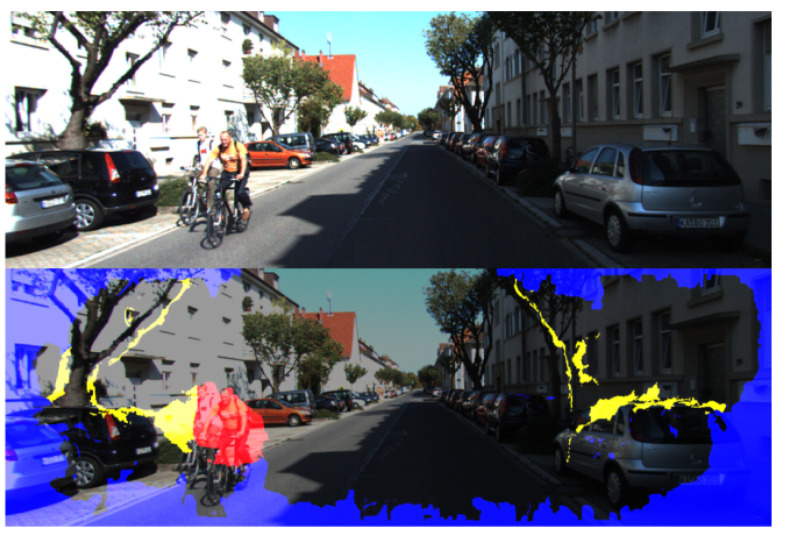
An example of a scene with the outlier region eliminated. The (**top**) scene is the original image while the (**bottom**) scene is the combination of the mask and image. According to the forward-backward consistency of the optical flow, all outlier regions are marked including occlusion regions (yellow), moving objects (red), boundaries and potential outliers (blue).

The forward flow at a non-occluded pixel should equal the inverse of the backward flow at the same pixel in the second frame. Based on this forward-backward consistency assumption, we used the accurate forward-backward OF fields generated by MaskFlownet to eliminate the outlier region in the scene. Specifically, when the condition is not satisfied, we flag pixels as potentially outliers. The constraint is formulated as:

(5) $${\left|F^{f}\left(p_{t}\right)+F^{b}\left(p_{t}+F^{f}\left(p_{t}\right)\right)\right|}^{2}<\alpha_{1}\left({\left|F^{f}\left(p_{t}\right)\right|}^{2}+{\left|F^{b}\left(p_{t}+F^{f}\left(p_{t}\right)\right)\right|}^{2}\right)+\alpha_{2}$$

where $F^{f}\left(p_{t}\right)$ denotes the forward flow of the pixel at $p_{t}$, $F^{b}\left(p_{t}\right)$ denotes the backward flow of the pixel at $p_{t}$. $\alpha_{1}$ and $\alpha_{2}$ were set to 0.01 and 0.5 in our experiment, respectively [26]. An example is shown in Figure 2, the generated mask effectively marks the invalid regions such as occlusion regions (yellow), moving objects (red) and boundaries (blue). In the boundary region, the backward OF cannot be calculated due to the camera’s moving, which results in inconsistent forward and backward OF. Then we impose the photometric consistency loss on the valid region, which is formulated as:

(6) $$L_{pho}=\underset{p_{t}\in V}{\sum }\rho \left(I_{t}\left(p_{t}\right)-{\bar{I}}_{t}\left(p_{t}\right)\right)$$

where *V* denotes the valid region.

### 3.4. Optical Flow Consistency Loss

The optical flow can be calculated from the scene depth $\left(D_{t}\right)$ and the relative camera pose $\left(T_{t\to t+1}\right)$ using 3D scene geometry. The calculated optical flow $\left(F^{cal}\right)$ can be represented by

(7) $$F^{cal}\left(p_{t}\right)=KT_{t\to t+1}D_{t}\left(p_{t}\right)K^{-1}p_{t}-p_{t}$$

Therefore, we can estimate the scene depth $\left(D_{t}\right)$ and the relative camera pose $\left(T_{t\to t+1}\right)$ to obtain the calculated optical flow through DepthNet and PoseNet, respectively. For non-occluded regions, the computed optical flow should be consistent with the generated forward optical flow (produced by MaskFlownet). Therefore, minimizing the difference between the two optical flow fields can be used as another loss function for jointly training DepthNet and PoseNet. Using the generated mask, our optical flow consistency loss is formulated as:

(8) $$L_{flo}=\underset{p_{t}\in V}{\sum }{\left||F^{cal}\left(p_{t}\right)-F^{f}\left(p_{t}\right)|\right|}_{1}$$

### 3.5. Depth Consistency Loss

According to the generated forward optical flow, the pixel mapping relationship of the consecutive images can be determined. Then, the relationship between the depth maps of the consecutive images can also be established. Therefore, we can synthesize a new depth map $\left({\bar{D}}_{t}\right)$ with the inverse warping from depth map $\left(D_{t+1}\right)$ by using the generated forward optical flow. The synthesized depth map $\left({\bar{D}}_{t}\right)$ and the target depth map $\left(D_{t}\right)$ should be consistent in the valid region. Consequently, we propose a depth consistency loss to train the DepthNet by penalizing the inconsistency between the synthesized depth map $\left({\bar{D}}_{t}\right)$ and the target depth map $\left(D_{t}\right)$. The depth consistency loss is formulated as:

(9) $$L_{dep}=\underset{p_{t}\in V}{\sum }{\left||D_{t}\left(p_{t}\right)-{\bar{D}}_{t}\left(p_{t}\right)|\right|}_{1}$$

## 4. Experiment and Results

We implemented our approach in the PyTorch platform and conducted all the experiments on a single NVIDIA GeForce GTX 1080Ti GPU with 11 GB memory. During training, the initial learning rate was set to 0.0002, the mini-batch was set to 4, and the loss weights were $\lambda_{s}=0.5,\;\lambda_{f}=0.2$ and $\lambda_{d}=0.2$. We used the Adam optimizer with β1 = 0.9 and β2 = 0.99. The images from the datasets were resized to 128 × 416 as the input of the network. Like other works [21,22,23], we applied several types of data augmentation methods to improve performance and prevent potential overfitting, including image color augmentation, rotational data augmentation and left–right pose estimation augmentation. Our model has approximately 25.28 million trainable parameters. The training typically converges after about 20 epochs. It took about 44.4 h to train the network. At testing, our model estimates depth and ego-motion with an average runtime of 14 ms and 63 ms per example.

### 4.1. Datasets and Metrics

Like the work in [16], we used the KITTI dataset [27] as our main training dataset, which is the largest and most commonly used dataset for autonomous driving applications such as VO, depth and optical flow, etc. The KITTI dataset provides 56 scenes of car driving and can be classified into “city”, “residential” and “road”. In addition, we used the Cityscapes dataset [28] to pre-train our model, which contains more than 50 cities’ stereo data without depth annotation. In order to evaluate the generalization ability of the network on a different dataset, we also used the Make3D dataset [29] in the testing phase. It only contains monocular images as well as corresponding depth maps and does not have monocular sequences and stereo image pairs.

Similar to other works such as [16,17,18,19,20,21,22,23], we used the absolute trajectory error (ATE) as the metric for pose estimation and we used the synthetic policy [30] as the metric for depth estimation. The ATE is defined as:

(10) $$F_{i}=Q_{i}^{-1}SP_{i},$$

(11) $$ATE=\sqrt{\frac{1}{N}\sum_{i=1}^{N}{\left||trans\left(F_{i}\right)|\right|}^{2}},$$

where $P_{i}$, $Q_{i}$ indicate the estimated pose value and its related ground truth. *S* denotes the similarity transformation matrix, and *trans* denotes fetching the translation part. The metrics used for depth estimation include the absolute relative error (*Abs. Rel*), the square relative error (*Sq. Rel*), the root mean squared error (*RMSE*), the log root mean squared error (*RMSE log*), and the prediction accuracy (δ). The definitions of these evaluation criteria are as follows:

(12) $$Abs.Rel=\frac{1}{N}\sum_{i=1}^{N}\frac{\left|D_{i}-D_{i}^{*}\right|}{D_{i}^{*}},$$

(13) $$Sq.Rel=\frac{1}{N}\sum_{i=1}^{N}\frac{{\left|D_{i}-D_{i}^{*}\right|}^{2}}{D_{i}^{*}},$$

(14) $$RMSE=\sqrt{\frac{1}{N}\sum_{i=1}^{N}{\left|D_{i}-D_{i}^{*}\right|}^{2}},$$

(15) $$RMSE\left(log\right)=\sqrt{\frac{1}{N}\sum_{i=1}^{N}{\left|lgD_{i}-lgD_{i}^{*}\right|}^{2}},$$

(16) $$\delta =max\left(\frac{D_{i}}{D_{i}^{*}},\frac{D_{i}^{*}}{D_{i}}\right)<T,$$

where $D_{i}$ and $D_{i}^{*}$ represent the estimated depth value and its related ground truth. For *T*, 1.25, 1.25^2^, and 1.25^3^ were used. The lower the value of the error metrics (*Abs. Rel*, *Sq. Rel*, *RMSE*, *RMSE log*) and the higher the value of the accuracy metric (δ), the better the performance. 

### 4.2. Ablation Study

We conducted an ablation study on different versions of the framework to investigate the effect of different components in our network. The baseline version was the model without outlier elimination, optical flow consistency loss and depth consistency loss. Since the DepthNet and the PoseNet are jointly trained, their accuracy is interdependent during training. Therefore, we only need to evaluate the effect on one of the two models. We conducted the experiments on KITTI odometry data, with 00–08 sequences used for training and 09–10 sequences utilized for testing. The results of the ego-motion evaluation are shown in Table 1. As indicated in the table, adding the outlier elimination, optical flow consistency loss and depth consistency loss can greatly enhance the model’s performance.

### 4.3. Evaluation of Ego-Motion Estimation

**Figure 3:**
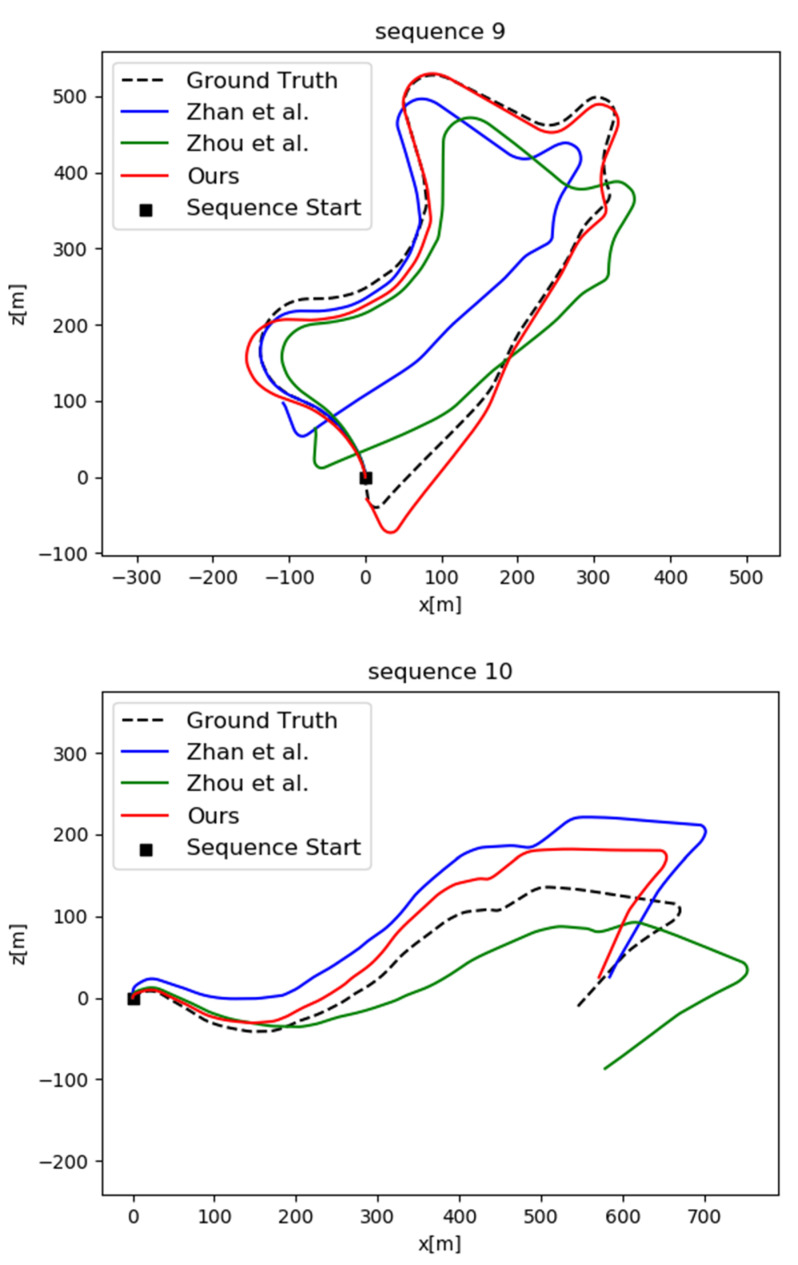
Comparison of trajectories produced by different methods on the KITTI sequence 09 and 10.

Although depth and pose are jointly trained, they are tested separately and their accuracy is interdependent. We evaluated the performance of PoseNet on the official KITTI visual odometry split, which includes 11 sequences with ground truth. We utilized 00–08 sequences for training and 09–10 sequences for testing to compare with other approaches. A row of each of the five images was truncated as a sequence of images as the input of the network during training. We compared our model to the other unsupervised methods [16,18,21,23] and the classical SLAM framework (ORB-SLAM). ORB-SLAM (full) allows for closed loop and re-localization, while ORB-SLAM (short) has no closed loop and re-localization. For the problem of scale ambiguity in monocular VO, we aligned the per-frame scale to the ground truth. The quantitative evaluation results for pose estimation on the KITTI dataset are shown in Table 2. The trajectories of sequence 09 and 10 produced by different methods are plotted in Figure 3. It is obvious that our method outperforms all of the others.

### 4.4. Evaluation of Depth Estimation

We used the KITTI dataset to evaluate the depth estimation. For better comparison with other works, we followed the dataset segmentation suggested by Eigen et al. [5] and Zhou et al. [16] for training and testing because the segmentation is commonly accepted by the research community for depth benchmark purposes. A total of 44,540 raw KITTI images were used for the training and validation, of which 40,109 images were used for training and 4431 for validation. The other 697 images were selected for testing. A row of each of the three images was truncated as a sequence of images as the input of the network during training. The ground truth was achieved by projecting the Velodyne laser scanned points onto the image plane for error and accuracy metrics evaluation. For the problem of scale ambiguity, we calculated a scale factor to match the estimated depth with the ground truth in the following form: $s=median\left(D_{gt}\right)/median\left(D_{pred}\right)$.

Table 3 shows the comparison between different studies. In the table, the second column defines the supervision signals used in the network. “Depth” means ground truth of depth and is used for supervised learning in the method, “Stereo” denotes that in the training, stereo sequences with known stereo camera pose are employed, and “Mono” means monocular sequences are used in the training. The third column defines the dataset used for training. K means trained only on KITTI dataset and CS + K denotes fine-tuning on the KITTI dataset following pre-training on the Cityscapes dataset. Our algorithm outperformed both the supervised and unsupervised methods, as demonstrated in Table 3.

Furthermore, we used the Cityscapes dataset [28] to pre-train our model and used the KITTI dataset for fine-tuning. The results (in the bottom part of Table 3) show some improvement in depth prediction, indicating that expanding the training data can enhance the models’ performance.

**Figure 4:**
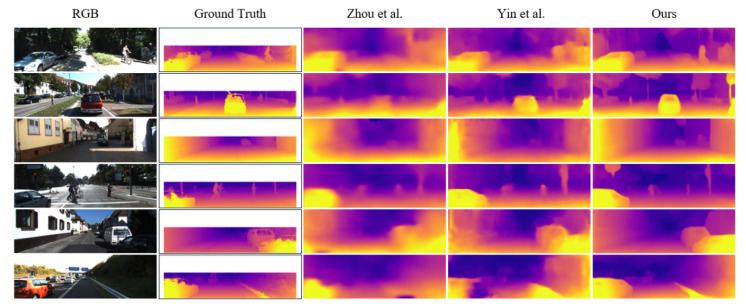
Depth examples of the unsupervised methods Zhou et al. [16], Yin et al. [21] and ours on KITTI dataset. The ground truth is interpolated for visualization.

The depth maps in Figure 4 were estimated by our model and in [16,21]. When compared to the other methods, our method produces sharper and more accurate depth maps.

### 4.5. Generalization to Other Datasets

**Figure 5:**
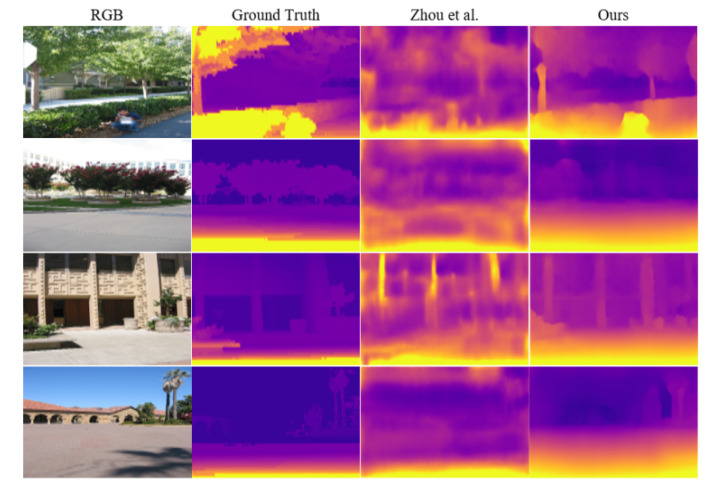
Depth estimation from the unsupervised methods of Zhou et al. [16] and ours on the Make3D dataset.

We tested the models on a new dataset, the Make3D without utilizing it for training to assess their generalization ability. In our experiment, we used the KITTI and Cityscapes datasets for training and used the Make3D for testing. The quantitative and qualitative evaluation results are shown in Table 4 and Figure 5. The results show that our method can perform well, even in unknown datasets and is superior to other methods.

## 5. Conclusions

We present a novel unsupervised learning pipeline for estimating depth and camera ego-motion in this study. We introduced a trained optical flow estimation network and made full use of it, including learning optical flow to estimate the camera motion, generating a mask that eliminates outlier regions, and adding additional geometric constraints. The results of the ablation experiments demonstrate their importance in improving the performance of the framework. Experiments on the KITTI dataset indicate that our algorithm outperforms other unsupervised methods. In the future, we want to enhance our framework to include a visual SLAM system to decrease drift.

## Figures and Tables

**Table 1:** Comparison of ATE of different versions of the framework on KITTI odometry. $L_{flo}$ and $L_{dep}$ indicate optical flow consistency loss and depth consistency loss, respectively.

Method	ATE of Seq.09	ATE of Seq.10
Baseline	0.017 ± 0.009	0.015 ± 0.010
Baseline + outlier elimination	0.012 ± 0.007	0.013 ± 0.007
Baseline + outlier elimination + $L_{flo}$	0.011 ± 0.007	0.010 ± 0.006
Baseline + outlier elimination + $L_{flo}$ + $L_{dep}$	0.010 ± 0.005	0.009 ± 0.006

**Table 2:** Comparison of ATE with different methods on KITTI VO dataset.

Method	ATE of Seq.09	ATE of Seq.10
ORB-SLAM (full) [2]	0.014 ± 0.008	0.012 ± 0.011
ORB-SLAM (short)	0.064 ± 0.141	0.064 ± 0.130
Zhou et al. [16]	0.016 ± 0.009	0.013 ± 0.009
Mahjourian et al. [18]	0.013 ± 0.010	0.012 ± 0.011
Yin et al. [21]	0.012 ± 0.007	0.012 ± 0.009
Ranjan et al. [23]	0.012 ± 0.007	0.012 ± 0.008
Ours	0.010 ± 0.005	0.009 ± 0.006

**Table 3:** Comparisons of different methods.

Method	Supervision Signal	Training Dataset	Error Metric				Accuracy Metric		
			*Abs.Rel*	*Sq.Rel*	*RMSE*	*RMSE (log)*	δ<1.25	$\delta <1.25^{2}$	$\delta <1.25^{3}$
Eigen et al. [5] Coarse	Depth	K	0.214	1.605	6.563	0.292	0.673	0.884	0.957
Eigen et al. [5] Fine	Depth	K	0.203	1.548	6.307	0.282	0.702	0.890	0.958
Liu et al. [6]	Depth	K	0.202	1.614	6.523	0.275	0.678	0.895	0.965
Zhan et al. [17]	Stereo	K	0.144	1.391	5.869	0.241	0.803	0.928	0.969
Godard et al. [30]	Stereo	K	0.148	1.344	5.927	0.247	0.803	0.922	0.964
Zhou et al. [16]	Mono	K	0.208	1.768	6.856	0.283	0.678	0.885	0.957
Zhou et al. [16] updated	Mono	K	0.183	1.595	6.709	0.270	0.734	0.902	0.959
Mahjourian et al. [18]	Mono	K	0.163	1.240	6.220	0.250	0.762	0.916	0.968
Yang et al. [19]	Mono	K	0.162	1.352	6.276	0.252	-	-	-
Yin et al. [21]	Mono	K	0.155	1.296	5.857	0.233	0.793	0.931	0.973
Ranjan et al. [23]	Mono	K	0.140	1.070	5.326	0.217	0.826	0.941	0.975
Godard et al. [30]	Mono	K	0.154	1.218	5.699	0.231	0.798	0.932	0.973
Ours	Mono	K	0.138	1.065	5.289	0.215	0.827	0.943	0.979
Zhou et al. [16]	Mono	CS + K	0.198	1.836	6.565	0.275	0.718	0.901	0.960
Mahjourian et al. [18]	Mono	CS + K	0.159	1.231	5.912	0.243	0.784	0.923	0.970
Yang et al. [19]	Mono	CS + K	0.159	1.345	6.254	0.247	-	-	-
Yin et al. [21]	Mono	CS + K	0.153	1.328	5.737	0.232	0.802	0.934	0.972
Ranjan et al. [23]	Mono	CS + K	0.139	1.032	5.199	0.213	0.827	0.943	0.977
Ours	Mono	CS + K	0.136	1.031	5.186	0.209	0.831	0.947	0.981

**Table 4:** Results of different methods on the Make3D dataset [29].

Method	Error Metric			
	*Abs. Rel*	*Sq. Rel*	*RMSE*	*RMSE (log)*
Liu et al. [6]	0.481	6.761	10.55	0.169
Zhou et al. [16]	0.396	5.731	10.869	0.513
Godard et al. [30]	0.579	11.235	11.892	0.201
Ours	0.301	3.367	8.142	0.261

## Data Availability

The data are available in a publicly accessible repository.
